# Object-mediated overwriting across saccades

**DOI:** 10.1167/jov.21.2.3

**Published:** 2021-02-04

**Authors:** A. Caglar Tas, J. Toby Mordkoff, Andrew Hollingworth

**Affiliations:** 1 Department of Psychology, University of Tennessee – Knoxville, TN, USA; 2 Department of Psychological and Brain Sciences, University of Iowa, IA, USA; 3 Department of Psychological and Brain Sciences, University of Iowa, IA, USA

**Keywords:** trans-saccadic perception, trans-saccadic integration, object-mediated updating, overwriting, saccades

## Abstract

How are visual sensory representations that are acquired peripherally from a saccade target related to sensory representations generated foveally after the saccade? We tested the hypothesis that, when the two representations are perceived to belong to the same object, the post-saccadic value tends to overwrite the pre-saccadic value. Participants executed a saccade to a colored target object, which sometimes changed during the saccade by ±15°, 30°, or 45° in color space. They were post-cued to report either the pre-saccadic or post-saccadic color in a continuous report procedure. Substantial overwriting of the pre-saccadic color by the post-saccadic color was observed. Moreover, the introduction of a brief post-saccadic blank interval (which disrupted the perception of object correspondence) led to a substantial reduction in overwriting. The results provide the first direct evidence for an object-mediated overwriting mechanism across saccades, in which post-saccadic values automatically replace pre-saccadic values.

## Introduction

Humans make frequent saccadic eye movements to extract detailed visual information from objects in the environment. Before a saccade, attention is shifted covertly to the saccade target object (e.g., Hoffman & Subramaniam, 1995), leading to the preferential encoding of the properties of that object (Irwin, 1992; Moore & Armstrong, 2003). When the eyes land, the visual system generates a foveal representation of the same object. What is the fate of the pre-saccadic representation, and how are the pre- and post-saccadic representations coordinated?

This basic question has a long and influential history in vision research. Early studies tested a *global sensory integration hypothesis* (McConkie & Rayner, 1976). Specifically, they tested whether low-level sensory patterns, generated pre- and post-saccadically, are spatiotopically fused to form a composite image. The hypothesized mechanism was equivalent to the high-capacity sensory fusion observed when two patterns are presented in rapid succession within a fixation, where the visible persistence of the first pattern (Coltheart, 1980) overlaps with sensory registration of the second pattern, producing the phenomenon of an integrated percept (Di lollo, 1977, 1980).^1^ However, several studies failed to observe such integration trans-saccadically (Bridgeman & Mayer, 1983; Irwin, 1991; Irwin, Yantis, & Jonides, 1983; O'Regan & Lévy-Schoen, 1983; Rayner & Pollatsek, 1983), indicating that high-capacity sensory fusion is not functional spatiotopically across saccades.

Recently, research on trans-saccadic perception has focused on the integration of surface feature properties of single objects, particularly the saccade target object, rather than the integration of global patterns (for reviews, see Aagten-Murphy & Bays, 2019; Herwig, 2015; Rolfs, 2015; Van der Stigchel & Hollingworth, 2018). Two lines of evidence indicate that local feature information can be integrated across saccades. First, when the pre- and post-saccadic target stimuli are the same, perceptual decisions are more precise than predicted from either source of information alone, a *reliability benefit* suggesting that the two sources of information were integrated at some stage before the perceptual decision (Ganmor, Landy, & Simoncelli, 2015; Hübner & Schütz, 2017; Stewart & Schütz, 2018a, 2018b, 2019; Wolf & Schütz, 2015). However, the locus and mechanism of integration in this paradigm are not fully understood. Second, when the pre- and post-saccadic stimuli *differ* on a dimension, such as two different colors, several studies have indicated that the two stimulus values are integrated to form an intermediate value (Demeyer, De Graef, Wagemans, & Verfaillie, 2010b; Ganmor et al., 2015; Oostwoud Wijdenes, Marshall, & Bays, 2015; Schut, Van der Stoep, Fabius, & Van der Stigchel, 2018; Wolf & Schütz, 2015). This would appear to reflect a relatively low-level, sensory locus of integration.

The latter example of integration may provide an explanation for one of the central phenomena that any account of trans-saccadic perception must address: insensitivity to trans-saccadic perceptual change (Bridgeman, Hendry, & Stark, 1975; Bridgeman & Stark, 1979; Grimes, 1996; Henderson & Hollingworth, 1999, 2003; McConkie & Currie, 1996). As an example of the basic effect, observers fail to detect surprisingly large spatial displacements of a saccade target when the shift is implemented during the saccade (Bridgeman et al., 1975; Bridgeman & Stark, 1979). This could simply reflect the fact that that the pre-saccadic information retained across the saccade is highly impoverished (e.g., O'Regan, 1992). However, Deubel, Schneider, and Bridgeman (1996) demonstrated that the limitation is not one of retention but rather of *access* to precise pre-saccadic information retained across the saccade. Deubel et al. (1996; see also Deubel, Bridgeman, & Schneider, 1998) modified the target displacement paradigm to include a brief blank period immediately after the eyes landed; that is, gaze landed on an empty display before the onset of the displaced target. Without a blank, discrimination of the direction of displacement was poor, replicating earlier studies. However, in the Blank condition, sensitivity to shift direction improved markedly. Precise spatial information was retained across saccade, but this was not available for comparison and report under normative conditions (i.e., without an artificial blank period). Similar effects have been observed for the retention of the surface feature properties of saccade targets (Grzeczkowski, Deubel, & Szinte, 2020; Grzeczkowski, van Leeuwen, Belopolsky, & Deubel, 2020; Poth & Schneider, 2016; Weiss, Schneider, & Herwig, 2015). Moreover, the effect of increased trans-saccadic sensitivity is not limited to blanking (and the resulting delay in target appearance) but generalizes to other changes that disrupt the continuity of the saccade target, such as polarity change (Tas, Moore, & Hollingworth, 2012).

The blanking effect indicates that trans-saccadic perception is typically characterized by a masking process when the pre- and post-saccadic properties of the target differ, impairing change discrimination. Tas et al. (2012) interpreted this as a form of object-based masking. When there is target continuity across the saccade (i.e., no blank), pre- and post-saccadic values are mapped to the same object representation, and the post-saccadic values mask the pre-saccadic values. However, when there is target discontinuity across the saccade (i.e., blank), the post-saccadic target is treated as a new object, the pre- and post-saccadic values are mapped to different object representations, and masking is reduced, supporting improved comparison and change discrimination.

In principle, there are two forms of visual masking and, thus, two possible masking mechanisms that could be functional in generating poor sensitivity to trans-saccadic change: integration masking and substitution masking. In the main literature on masking within a fixation (for reviews, see Breitmeyer & Ogmen, 2006; Enns & Di Lollo, 2000), integration masking is observed at very short target–mask stimulus-onset asynchrony (SOA) up to approximately 100 ms. As the name implies, one perceives an integrated stimulus. For example, if two different color stimuli are presented in rapid succession, participants perceive a single, intermediate hue, impairing access to the component values (Efron, 1967; Pilz, Zimmermann, Scholz, & Herzog, 2013). For longer SOAs, one tends to observe substitution masking (sometimes termed *interruption* masking), in which only the masking stimulus is perceived. That is, the mask tends to overwrite or replace the representation of the target. Either or both might be functional across saccades, perhaps depending on variables such as the magnitude of trans-saccadic change or the relative precision of the pre- and post-saccadic representations (Atsma, Maij, Koppen, Irwin, & Medendorp, 2016).

Demeyer et al. (2010b) provided evidence to support a trans-saccadic integration masking mechanism. They manipulated the aspect ratio of a saccade target ellipse across the saccade, with the ellipse changing to become more circular or less circular. Participants were asked to report only the post-saccadic value by selecting that value from eight possible values in a linear array. This task allowed them to test for automatic effects of the pre-saccadic value on perception of the post-saccadic value. The key finding was that the mean of the response distribution was shifted to a value intermediate between the pre- and post-saccadic values, suggesting automatic feature integration that impaired access to the post-saccadic shape (for similar results, see Oostwoud Wijdenes et al., 2015; Schut et al., 2018). Critically, the introduction of a blank period after the saccade decreased the bias, suggesting that integration was dependent on target continuity.

In contrast, Tas et al. (2012) considered substitution as the more plausible form of trans-saccadic masking, because integration masking is functional only at SOAs shorter than those typically observed trans-saccadically. Moreover, they treated object-based trans-saccadic masking as an example of a general visual mechanism termed *object-mediated updating* (Tas et al., 2012; see also Moore & Enns, 2004; Moore & Lleras, 2005; Moore, Mordkoff, & Enns, 2007). Specifically, several phenomena indicate that changes to the state of a single perceptual object lead to the replacement of the representation of the previous state with the subsequent state. This updating occurs in phenomena as diverse as motion de-blurring (Moore, Mordkoff, & Enns, 2007), object substitution masking (Moore & Lleras, 2005), and the flash-lag effect (Moore & Enns, 2004). Although substitution is a plausible form of trans-saccadic masking, there is currently no direct evidence to support it. That is, there is currently no empirical result indicating that post-saccadic stimulus values overwrite pre-saccadic values when they are mapped to the same object representation. The goal of the present study was to provide such evidence.

## Present study

**Figure 1.**
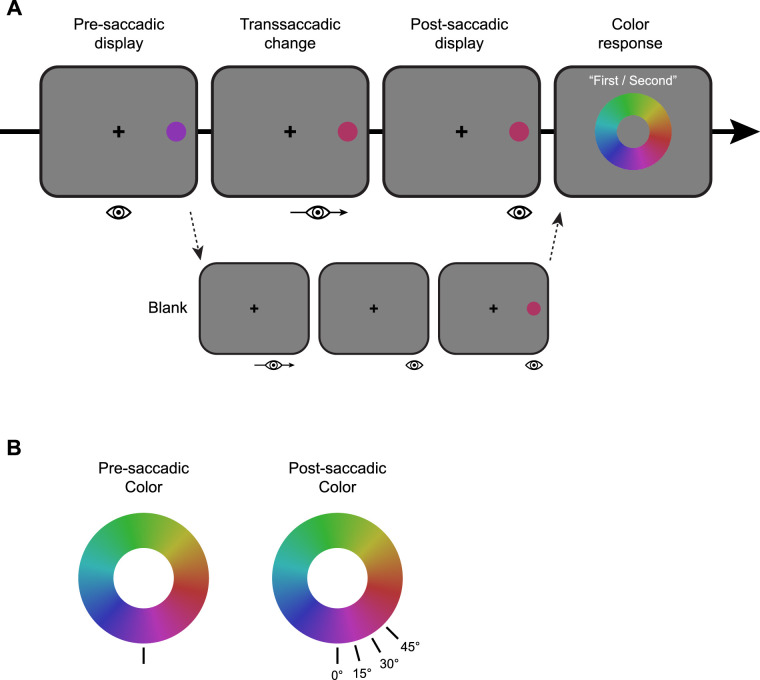
(A) Sequence of events in a trial. The top row shows the sequence of events in the No-blank condition. For Blank trials, the screen was blanked for 250 ms after detection of the saccade. The eye icon represents participants’ eye position at each stage of the trial. Note that stimuli are not drawn to scale. (B) The color of the post-saccadic disk was either the same as the pre-saccadic disk (0°) or shifted ±15°, 30°, or 45° in color space.

The basic paradigm is illustrated in Figure 1. The method was similar to that used by Demeyer et al. (2010b). A single, colored saccade target appeared to the left or right of central fixation. During the saccade to the target, the color either remained the same or was changed 15°, 30°, or 45° in color space. In addition, a post-saccadic blank period either was or was not introduced. After presentation of the post-saccadic target, participants were cued to report either the pre-saccadic or the post-saccadic target color by selecting the appropriate color on a color wheel.

There are several aspects of this design upon which to elaborate. First, instead of manipulating the aspect ratio (Demeyer et al., 2010b), we manipulated the color of the saccade target (Oostwoud Wijdenes et al., 2015; Schut et al., 2018). The use of a circular dimension significantly simplifies the interpretation of the resulting response distribution. A bounded dimension like the aspect ratio can introduce response biases, such as a bias to avoid reporting extreme values (Demeyer et al., 2010b).

Second, we had participants report either the pre- or post-saccadic value rather than just the post-saccadic value, as in Demeyer et al. (2010b). This method was necessary to test the assumption that the post-saccadic value would tend to overwrite the pre-saccadic value, rather than vice versa, which requires collecting color response distributions for the report of both pre- and post-saccadic values. This method also encouraged participants to maintain separate pre- and post-saccadic representations rather than integrating them into a single, merged representation, a necessary condition for observing possible overwriting. Specifically, automatic overwriting of the pre-saccadic value by the post-saccadic value is best tested in the context of a demand to preserve the pre-saccadic value.

**Figure 2.**
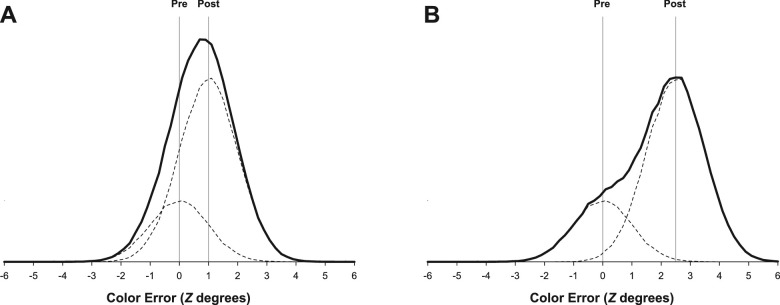
Simulated distributions reflecting a mixture of responses centered at the pre- and post-saccadic values when participants were cued to report the pre-saccadic color. The aggregate distributions (solid lines) are composed of two “basis” distributions (dashed lines), with 75% of responses drawn from a distribution centered at the post-saccadic color and 25% from a distribution centered at the pre-saccadic color (i.e., substantial overwriting). (A) The graph illustrates a distributional separation of d’ = 1. (B) The graph illustrates a distributional separation of *d´* = 2.5.

Finally, one of the key challenges here is the need to distinguish between a *bivariate response distribution* that reflects a mixture of responses centered at the pre- and post-saccadic values and a *univariate response distribution* centered at a value intermediate between the pre- and post-saccadic values. The former allows examination of possible overwriting, reflected in the relative probability of pre-saccadic versus post-saccadic color report. The latter does not, because it would instead be consistent with a different mechanism of masking: namely, integration. To illustrate the problem, consider a condition in which participants are cued to report the pre-saccadic color (Figure 2). Hypothetically, 75% of responses are drawn from a distribution centered, instead, at the post-saccadic color (i.e., substantial overwriting) and 25% from a distribution centered at the correct, pre-saccadic color. If the two distributions are separated by *d´* = 1 (Figure 2A), then the combined response distribution will be very difficult to distinguish from a univariate distribution centered at an intermediate value, especially because the bivariate mixture is unimodal.^2^ There are two ways to improve the ability to discriminate between univariate and bivariate structures in the aggregate response distribution: 1) equalize the probabilities of responses from the two distributions and 2) increase the distributional separation (Yantis, Meyer, & Smith, 1991). The latter is more tractable in the present context. Figure 2B illustrates the same mixture of responses but for pre- and post-saccadic colors that are instead separate by *d´* = 2.5. The underlying bivariate structure becomes more apparent. To address this issue formally, as a first step to our tests of overwriting, we fit univariate and bivariate mixture models (Bays, Catalao, & Husain, 2009; Zhang & Luck, 2008) to the observed response distributions to ensure that the data were more likely to have been generated by a bivariate response structure than by a univariate structure. In addition, we used two relatively large change magnitudes (30° and 45°, in addition to 15°) to increase the distributional separation and thus maximize our ability to distinguish between the two response structures. Larger change magnitudes are also likely support the maintenance of separate representations rather than an integrated representation (Atsma et al., 2016), because discrepancies should be easier to detect, again establishing conditions amenable to observing overwriting rather than integration.

The object-mediated updating hypothesis (Tas et al., 2012) makes the broad prediction that we should observe overwriting of the pre-saccadic color value by the post-saccadic value, particularly under circumstances that support the perception of a single object trans-saccadically. Specifically, this hypothesis predicts the following pattern of results. First, under conditions of target continuity (no blank), when participants are cued to report the pre-saccadic color, there should be a substantial proportion of trials on which they erroneously report the post-saccadic color (i.e., an overwriting effect). Second, because overwriting is proposed to be directional (newer states overwrite older states but not vice versa), when cued to report the post-saccadic color, there should be minimal erroneous report of the pre-saccadic color. Third, the overwriting effect for pre-saccadic color report should be decreased when a blank period is introduced, because the two colors will be mapped to separate object representations, and the pre-saccadic value should be protected from object-based overwriting. Finally, the absolute magnitude of overwriting should diminish with increasing color change magnitude, as larger surface feature discontinuities should also lead to the perception of two different objects (Demeyer, De Graef, Wagemans, & Verfaillie, 2010a; Tas et al., 2012).

## Methods

### Participants

A total of 71 participants (18–30 years of age, 44 female) from the University of Tennessee Knoxville community completed the experiment. They received course credit for their participation. All human subjects procedures were approved by the University of Tennessee Knoxville Institutional Review Board. Prior to the experiments, we conducted power analyses to determine sample size with MorePower 6.0.1 (Campbell & Thompson, 2012). The effect size was taken from a pilot study with 45° color change. With an observed effect size of η_p_^2^ = .405, a minimum of 18 participants is needed to achieve .90 power for a 2 × 2 × 2 repeated-measures design. In all experiments, we targeted a minimum sample size of 20 to ensure sufficient power. The change magnitude was manipulated between participant groups (22 participants at 15°, 25 at 30°, and 24 at 45°). All participants reported normal or corrected-to-normal vision and were tested for color blindness with the eight-plate version of the Ishihara color deficiency test. Two participants in the 15° sub-experiment and two in the 30° sub-experiment were eliminated because their mean saccadic reaction times were more than 2 standard deviations (*SD*) above the group mean (a criterion established a priori on the basis of the pilot study).^3^ Two participants in the 45° sub-experiment were eliminated due to issues during the experiment (one for computer failure and the other for being provided incorrect instructions). Thus, 20, 23, and 22 participants were included for the 15°, 30°, and 45° sub-experiments, respectively.

### Stimuli and apparatus

All stimuli were presented on a grey background with a central, black fixation cross subtending 0.46 × 0.46 degrees of visual angle (dva). The pre- and post-saccadic objects were colored disks subtending 0.33 dva. The saccade- target disk was presented either to the left or right of central fixation (randomly selected) at an eccentricity randomly selected within the range of 5.0 to 7.0 dva. The pre-saccadic color value was randomly chosen at the beginning of each trial from a set of 360 possible colors equally distributed in HSV color space, with the saturation and value (lightness) parameters held constant at 0.7.

On No-change trials, the disk had the same color value pre- and post-saccadically. On Color-change trials, the color of the post-saccadic disk was changed 15°, 30°, or 45° in color space, with a clockwise or counterclockwise direction chosen randomly. The critical data came from Color-change trials. No-change trials were included to decrease the probability that participants would infer that there was a color discrepancy of an equivalent magnitude on every trial.

The color wheel used to collect responses was an annulus with an outer radius of 7 dva and an inner radius of 3 dva, presented centrally. To eliminate spatial response biases, the orientation of the color wheel was chosen randomly on each trial. The text instructing participants to report either the pre-saccadic (“Report First Color”) or post-saccadic (“Report Second Color”) color was black and was presented 1.6 dva above the color wheel.

In all experiments, stimuli were displayed on an LED monitor with 1280 × 960 resolution and a refresh rate of 100 Hz. The position of the right eye was monitored by an SR Research Eyelink 1000 Plus eye tracker sampling at 1000 Hz. A chin and forehead rest maintained a viewing distance of 77 cm and minimized head movements. Stimulus presentation was controlled with E-prime software (Schneider, Eschmann, & Zuccolotto, 2002).

### Experimental design and procedure

All three change-magnitude sub-experiments had a 2 (Color Change: No-change, Color-change) × 2 (Blanking: No-blank, Blank) × 2 (Reported Color: Pre-report, Post-report) within-subjects design, with trials from the individual conditions randomly intermixed.

The events on a trial and key manipulations are illustrated in Figure 1. The experimenter initiated each trial after visual confirmation of central fixation. After a random delay of between 1000 and 1500 ms, the pre-saccadic disk was presented. Participants were instructed to execute a saccade to the disk as quickly as possible. If the participant's gaze deviated more than 1.5 dva from the central fixation point before the disk appeared, a red screen was presented indicating a gaze error, and that trial was aborted and added back into the trial pool. This occurred on 5% of the trials for the 15°-change sub-experiment, 8% for 30°, and 10% for 45°.

The trans-saccadic change was implemented using a boundary technique. After the presentation of the target disk, the computer monitored for an eye position sample more than 2.0 dva from central fixation in the direction of the disk. Upon detection, the color change was implemented within a maximum of 10 ms, ensuring that the change was completed before the end of the saccade. For trials in the Blank condition, the disk was removed for 250 ms after the eye tracker detected the boundary crossing, and the post-saccadic disk was then written to the screen. After the start of the post-saccadic disk display, the computer monitored for an eye sample within 1.0 dva from the post-saccadic disk.

Upon detection, the post-saccadic disk remained on the screen for a variable duration. This duration was equal to the saccadic reaction time of the previous trial (disk onset to boundary crossing), approximately equating the exposure durations of the pre- and post-saccadic disks across the experiment. (We used the saccade latency of the previous trial, rather than the current trial, to minimize the possibility that participants would delay saccade initiation in order to lengthen the exposure duration of both the pre- and post-saccadic color.) If the previous trial latency was less than 150 ms or more than 700 ms, a fixed value of 260 ms was used for the post-saccadic display duration (the mean latency value observed in pilot work). In addition, the post-saccadic duration was set to 260 ms for the first trial of the practice block and the first trial of the experimental block.

Finally, the color wheel was presented centrally with instructions to report either the pre- or post-saccadic color. Participants responded by using a mouse to click the appropriate value on the color wheel.

Participants first completed eight practice trials that were not included in the analyses. For the experimental block, they completed 480 trials, 60 in each of the eight conditions (defined by Change vs No-change, Blank vs No-Blank, and Pre-report vs Post-report), randomly intermixed. The experiment lasted approximately 90 minutes.

### Analyses and model fitting

In these analyses, participants’ color response distributions were fit with probabilistic mixture models (e.g., Bays et al., 2009; Zhang & Luck, 2008). The first step was to ensure that the results were more likely to have been generated by a bivariate response structure than by a univariate structure. We then used the probability estimates from the bivariate model to quantify the proportion of responses corresponding to the target and distractor distributions.

A bivariate *dual-gaussian model* implemented the hypothesis that the full response distribution reflected subsets of responses centered at the target value and at the distractor value:

(1) $$\;p\left(x\right)=p_{t}\phi_{\mu_{t},\kappa }\left(x-\theta_{t}\right)+\;p_{d}\phi_{\mu_{d},\kappa }\left(x-\theta_{d}\right)+p_{r}/2\pi$$

where *x* refers to the reported value, θ_*t*_ to the actual value of the target disk, and θ_*d*_ to the distractor value. The target value corresponded either to the pre-saccadic value (on Pre-report trials) or to the post-saccadic value (on Post-report trials). The reported color value was calculated as a difference score from the target value (thus, target color was always represented at 0°). Difference scores were normalized so that deviations toward the distractor color value (at 15°, 30°, or 45°) were positive. Each of the two gaussians ($\phi_{\mu_{t},\kappa }$ and $\phi_{\mu_{d},\kappa }$) constituted a probability density function of a von Mises distribution with fixed means at the target (*µ_t_*) and distractor (*µ_d_*) values and a concentration of *κ* ($SD=\;\sqrt{1/\kappa }$). Note that the equivalent concentration for the two gaussians was a simplifying assumption that allowed us to equate the number of free parameters between models. Last, *p_t_*, *p_d_*, and *p_r_* refer to probabilities of reporting the target color, the distractor color, or a random color value, respectively [*p_t_* = 1 −  (*p_d_* + *p_r_*)]. The model had three free parameters: *κ*, *p_d_*, and *p_r_*. This model is equivalent to the *swap model* of Bays et al. (2009), but applied to an experimental design in which the distractor value was fixed at one of the three change magnitudes (rather than chosen randomly on each trial).

A *single-gaussian model with* *a* *variable mean* was used to implement the hypothesis that the pre- and post-saccadic colors were perceptually integrated to generate an intermediate color representation:

(2) $$p\left(x\right)=p_{t}\phi_{\mu_{t},\kappa }\left(x-\theta_{t}\right)+p_{r}/2\pi$$

This model also had three free parameters: *µ_t_*, *κ*, and *p_r_*. Treating the mean of the target distribution as a free parameter allowed the model to fit the potential shift of the distribution to an intermediate value. This model is sometimes referred to as a *standard mixture model with bias* (Suchow, Brady, Fougnie, & Alvarez, 2013).

Maximum likelihood estimates of the parameters for each model were calculated for each participant with MatLab's *mle* function, which uses the non-linear optimization procedure (*fminsearch* function) developed by Nelder and Mead (1965). The model fitting was implemented using code adapted from the MemToolbox (Suchow et al., 2013). A range of start values was used to avoid local minima.

### Eye-tracking data analysis and trimming

An Eye-tracking data analysis was conducted offline. A combined velocity (30°/s) and acceleration (8,000°/s^2^) threshold was used to define saccades. Trials were eliminated from the analysis if saccade latency was shorter than 100 ms or longer than 550 ms, or if the eye failed to land within 1.5 dva from the target. A total of 13% of trials at 15° color change, 10% at 30° color change, and 10% at 45° color change were excluded from the analyses.

After trimming, the mean latency of the saccade to the target disk was 204 ms (*SD* = 75 ms) for 15° color change, 210 ms (*SD* = 78 ms) for 30° color change, and 206 ms (*SD* = 71 ms) for 45° color change. After the onset of the saccade, the change-triggering boundary was crossed within approximately 20 ms, on average, with mean elapsed time to change initiation of 223 ms (*SD* = 75 ms) for the 15° change, 230 ms (*SD* = 84 ms) for the 30° change, and 226 ms (*SD* = 72 ms) for the 45° change. We did not analyze saccade latency as a function of condition, because all within-subject manipulations were implemented after the saccade had been launched.

## Results

**Figure 3.**
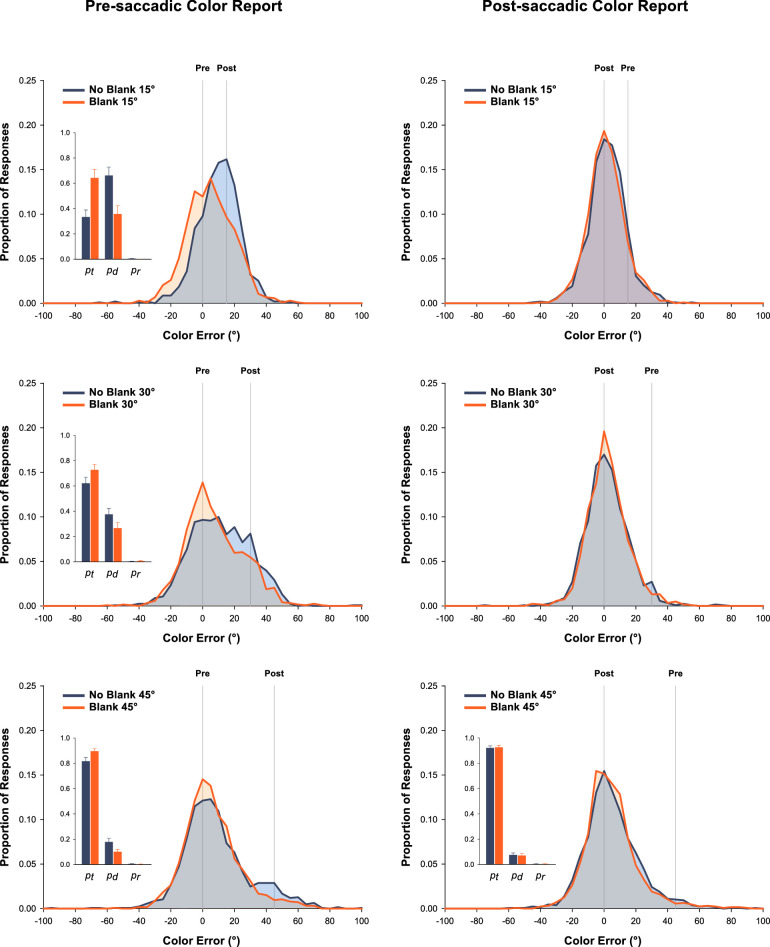
Color response distributions for the Pre-report (left) and Post-report (right) trials as a function of color change magnitude and blanking. The data were normalized for report so that the correct value is displayed at zero and the distractor value at 15°, 30°, or 45°. Parameter estimates derived from the dual-gaussian model are inset. The parameters *p_t_*, *p_d_*, and *p_r_* reflect the probabilities of reporting the target color, the distractor color, or a random color value, respectively. Error bars are standard errors of the means.

Figure 3 shows the response distributions for Pre-report trials (left column) and Post-report trials (right column) for the No-blank condition (drawn in dark blue) and the Blank condition (drawn in orange). Results from the three change-magnitude sub-experiments are reported separately. Response distributions for the no-change trials are reported in supplemental materials.

### Pre-report trials

The Pre-report trials in the No-blank condition were central to the analysis, because it was in this condition that we predicted substantial overwriting. We first fit the dual-gaussian (equation 1) and single-gaussian (equation 2) models to these data. At each of the three change magnitudes, response distributions were more consistent with the bivariate model than with the univariate model (Table 1), in accordance with our assumption that the structure of this experiment would lead to the maintenance of separate representations rather than an integrated representation. Moreover, evidence in support of the dual-gaussian model increased with increasing change magnitude, consistent with greater power to distinguish between models with larger distributional separation (Figure 2).

**Table 1.** Parameter estimates and goodness-of-fit metrics for the single-gaussian with bias model (model 1) and the dual-gaussian model (model 2) for the No-blank trials at each of the three change magnitudes. *Notes*: The models were fit to each participant's data, and reported parameter estimates are means of the participant estimates. For the single-gaussian model, there were three free parameters: µ (mean of gaussian distribution), κ (concentration of gaussian distribution, reported as $SD=\sqrt{1/\kappa }$), and *p_r_* (probability of a response from the uniform distribution, i.e., a random guess). We also report *p_t_* (probability of report from gaussian distribution), which was derived from *pr*, (*p_t_* = 1 − *p_r_*). For the dual-gaussian model, the means of the two gaussians were fixed at the target and distractor values. There were also three free parameters: κ, *p_d_* (probability of response from the distractor distribution), and *p_r_*. Again, *p_t_* was derived [*p_t_* = 1 − (*p_a_* + *p_r_*)]. For each model, mean log(*L*) and mean Bayes information criterion (BIC) are reported. For model comparison, Δ(BIC) = BIC_model1_ − BIC_model2_, with positive values indicating a superior fit to the dual-gaussian model.

	Single gaussian with bias (model 1)						Dual gaussian (model 2)						
Condition	µ	*SD*	*pr*	*Pt*	Log (*L*)	BIC	*SD*	*pa*	*pr*	*Pt*	Log (*L*)	BIC	Δ (BIC)
Pre-report 15°	10.14°	10.25°	0.004	0.996	−201.8	415.5	10.25°	0.662	0.005	0.333	−201.7	415.2	0.26
Pre-report 30°	11.23°	17.26°	0.004	0.995	−228.8	469.5	12.62°	0.375	0.003	0.622	−228.1	468.2	1.32
Pre-report 45°	8.68°	19.44°	0.009	0.991	−233.5	478.9	14.10°	0.178	0.005	0.817	−232.2	476.3	2.66
Post-report 15°	2.14°	11.44°	0.005	0.995	−202.3	416.4	10.38°	0.129	0.005	0.865	−202.4	416.7	−0.24
Post-report 30°	2.09°	12.35°	0.007	0.993	−228.8	429.8	11.23°	0.052	0.005	0.942	−228.1	430.3	−0.45
Post-report 45°	4.86°	15.55°	0.016	0.984	−228.0	468.0	13.91°	0.075	0.003	0.921	−227.8	467.6	0.42

Next, we used the estimates of *p_t_* (probability of target report) and *p_d_* (probability of distractor report) from dual-gaussian fits in the No-blank condition to assess the extent to which the post-saccadic values overwrote the pre-saccadic values. These data are inset within the panels of Figure 3 (drawn in dark blue). The probability of erroneous report of the post-saccadic (distractor) value was .662 at 15°, .375 at 30°, and .178 at 45°. Note that, at 15°, the majority of responses at the distractor value and the small distributional separation led to the appearance of a shift in the entire distribution toward the post-saccadic value. Overall, then, we observed substantial overwriting in the No-blank condition. In addition, distractor report probability decreased reliably with increasing change magnitude, *F*(2,64) = 29.4, *p* < .001, adj $\eta_{p}^{2}$ = .463. Note that we report the adjusted $\eta_{p}^{2}$, which removes the positive bias inherent in standard $\eta_{p}^{2}$ (Mordkoff, 2019).

Finally, we obtained estimates of *p_t_* and *p_d_* for the Blank trials in the Pre-report condition (orange bars inset in the panels of Figure 3). This method allowed us to test the prediction that unambiguous object discontinuity would lead to protection of the pre-saccadic color from overwriting (Deubel et al., 1996; Poth & Schneider, 2016; Tas et al., 2012) and thus increase the proportion of correct, pre-saccadic responses. We entered the *p_t_* data into a 2 (Blank, No-blank) × 3 (Change magnitude) mixed factor analysis of variance. There was a reliable main effect of Blanking, *F*(1,62) = 47.4, *p* < .001, adj $\eta_{p}^{2}$ = .424, with the probability of target report reliably higher in the Blank condition than in the No-blank condition. There was also a reliable main effect of Change Magnitude, *F*(1,62) = 21.2, *p* < .001, adj $\eta_{p}^{2}$ = .397, with the probability of target report increasing with increasing change magnitude. Finally, there was a reliable interaction, *F*(2,62) = 8.86, *p* < .001, adj $\eta_{p}^{2}$ = .197, with the benefit of blanking on the target report most pronounced in the 15° sub-experiment and systematically decreasing with increasing color change. This interaction was due, presumably, to the fact that the 15° condition had the smallest probability of target report in the No-blank condition (i.e., the greatest probability of overwriting) and thus the greatest potential for improvement with blanking. Nevertheless, the effect of blanking was statistically reliable at all three change magnitudes [15°: *t*(19) = 4.79, *p* < .001, adj $\eta_{p}^{2}$ = .523; 30°: *t*(22) = 3.40, *p* = .003, adj $\eta_{p}^{2}$ = .314; 45°: *t*(21) = 3.59, *p* = .002, adj $\eta_{p}^{2}$ = .350]. Given that there were very few guesses in these experiments (the *p_r_* was always close to zero), an analysis over the *p_d_* data produced complementary results.

### Post-report trials

The object-mediated updating hypothesis posits that subsequent states of an object tend to overwrite previous states of an object (and not vice versa), and thus we predicted that there would be minimal erroneous reports of the pre-saccadic value when cued to report the post-saccadic value. As is evident in the distributions displayed in the right column of Figure 3, this was indeed the case, with the large majority of responses concentrated at the correct, post-saccadic value. With this pattern, we had very limited power to detect evidence for an underlying bivariate structure in these data, because sensitivity to multinomial mixtures depends on both separation (between the “basis” distributions) and the mixing parameter (i.e., the proportion of trials from each).

After fitting the bivariate and univariate mixture models to the Post-report, No-blank data, only the results from the 45° sub-experiment were better fit by the bivariate model (Table 1), and thus only at this change magnitude did we draw conclusions from analyses concerning potential overwriting. For the 45° sub-experiment, we calculated the proportion of responses corresponding to the target value (*p_t_* = .921) and to the distractor value (*p_d_* = .075). The probability of target report was reliably higher in this No-blank, Post-report condition than in the corresponding No-blank, Pre-report condition at 45° change (*p_t_* = .817), *t*(21) = 4.11, *p* < .001, adj $\eta_{p}^{2}$ = .420. That is, there was a higher probability that the post-saccadic color was erroneously reported than that the pre-saccadic color was erroneously reported, consistent with the hypothesis that more recent states of an object tend to overwrite earlier states of an object. We also calculated *p_t_* and *p_d_* for the Blank, Pre-report trials at 45° change (*p_t_* = .926; *p_d_* = .071). For the *p_t_* data, there was no increase in correct, Post-saccadic report in the Blank condition compared with the No-blank condition, *t*(21) = .34, *p* = .732, adj $\eta_{p}^{2}$ = –.041. That is, there was no blanking effect for the Post-report trials, in contrast with the robust blanking effect observed for the Pre-report trials at this change magnitude.^4^ Two caveats apply to this analysis. First, the evaluation of a blanking effect for Post-report trials may have been limited by near-ceiling probability of target report. Second, the present design cannot eliminate an alternative hypothesis that higher quality perceptual information (here, post-saccadic, foveal information) preferentially overwrites lower quality perceptual information, such that the effect might have been reversed if participants had made a saccade away from the tested object. Nevertheless, the absence of overwriting in the post-report trials is consistent with our assumption that blanking protects the pre-saccadic color from being overwritten by the post-saccadic color, but not necessarily vice versa.

## Discussion

In the present study, we tested an object-mediated updating account of how pre- and post-saccadic representations of an object are related to each other (Tas et al., 2012). In this view, the post-saccadic value of a target will tend to overwrite the pre-saccadic value when the two are perceived to be properties of a single, continuous object. We tested four specific predictions. First, when it was likely that pre- and post-saccadic features were mapped to the same object representation, overwriting of the pre-saccadic value by the post-saccadic value should have been observed. This was indeed the case. In the Pre-report, No-blank condition, participants erroneously reported the post-saccadic color on a substantial proportion of trials (almost two-thirds of trials in the 15° change condition), despite the fact that they had a strong incentive to preserve the pre-saccadic color. This finding is the most direct demonstration to date of the basic overwriting phenomenon. Second, because object-based substitution masking is directional, there should have been minimal erroneous report of the pre-saccadic color when cued to report the post-saccadic color. This prediction was also observed. Third, when object continuity was disrupted by a blank period (Deubel et al., 1996), the pre- and post-saccadic features should have been mapped to different object representations, protecting the pre-saccadic features from overwriting. Consistent with this prediction, the introduction of a blank period after the saccade led to substantial improvement in pre-saccadic color report. This is clear evidence of *protection from overwriting* based on object discontinuity. Finally, because substantial surface feature differences can also disrupt object continuity (Demeyer et al., 2010a; Tas et al., 2012), the probability of overwriting, and the resulting improvement with blanking, should also have decreased with increasing color change magnitude, and this pattern was likewise observed. Taken together, the present results constitute some of the most direct evidence to date that relatively precise pre-saccadic information is retained across saccades, but is rendered unavailable for report under typical, no-blank conditions (Deubel, Bridgeman, & Schneider, 1998; Deubel et al., 1996; Poth & Schneider, 2016). Most important, it is the first evidence that overwriting plays a key role in how pre- and post-saccadic information are coordinated.

This brings us back to the question of why changes across saccades are difficult to detect. As discussed above, there are two main possibilities. Changes may be difficult to detect because post-saccadic feature values overwrite pre-saccadic values, precluding comparison (Tas et al., 2012). Additionally, the pre- and post-saccadic values may be merged to form an intermediate value, also precluding comparison (Demeyer et al., 2010b). The present data provide evidence for the former mechanism. Support for the latter mechanism comes from three studies that have used the continuous report technique to show that, when different values of a target are presented pre- and post-saccadically, the mean of the response distribution shifts to an intermediate value (Demeyer et al., 2010b; Oostwoud Wijdenes et al., 2015; Schut et al., 2018). This shift has been interpreted as indicating that the two representations were merged to form a single, intermediate representation. Two of these used color as the relevant dimension (Oostwoud Wijdenes et al., 2015; Schut et al., 2018), as here, and thus we focus on these studies in our discussion.

In each of these studies, the presence of a unimodal response distribution was interpreted as reflecting an underlying univariate response structure (a single response distribution centered at an intermediate value). However, unimodality is not a sufficient condition for this inference, because an underlying bivariate structure (e.g., two component distributions centered at each of the original values) can merge to produce a unimodal pattern if there is insufficient separation between the component distributions (Figure 2A). Because we were interested in overwriting rather than integration, as a first step to the analysis, we obtained evidence that the response structure was more likely to have been bivariate than univariate. The same requirement applies to studies that would draw conclusions based on the assumption that the response distribution is univariate, reflecting a merged representation (Demeyer et al., 2010b; Oostwoud Wijdenes et al., 2015; Schut et al., 2018), but equivalent tests were not conducted for these experiments to ensure that the data were indeed more likely to have been generated by a univariate response structure. To highlight the issue, consider that, in the present study, we typically observed unimodal response distributions, and if we had simply calculated the means of these distributions, we would have observed a “shift” toward an intermediate value very similar to those observed in the three studies discussed here. Yet, the underlying response structure was often more consistent with a bivariate mixture of responses centered at the two original values than with a single response distribution centered at an intermediate value. Thus, the inferential strength of the studies proposing a merged representation (Demeyer et al., 2010b; Oostwoud Wijdenes et al., 2015; Schut et al., 2018) is contingent on the type of formal mixture analysis used here, which could be applied post hoc to existing data (if the designs have sufficient power to detect a possible bivariate mixture).^5^

If we consider that these earlier studies did indeed reflect relatively low-level sensory integration to form an intermediate value, what are the key factors that may have caused us to observe overwriting of separate representations, instead? The magnitude of color change is a candidate, as larger change magnitudes might decrease the probability or extent of integration (Atsma et al., 2016). However, this is unlikely to have been a contributing factor. Oostwoud Wijdenes et al. (2015) implemented a 20° color change, and Schut et al. (2018) implemented a 30° color change, both of which fall within the range of color changes used here. In addition, it is helpful to contextualize absolute color-change magnitudes by the variability in the response distribution. Here, the SD for report of the pre-saccadic color ranged from 10° to 14° (estimated from the dual-gaussian fit to the Pre-report, No-blank data; see Table 1). Thus, our smallest change magnitude of 15° was approximately 1.0 to 1.5 times the SD. This SD estimate is similar to the 12° to 16° SD observed in Stewart and Schütz (2018b) when only a single, pre-saccadic color was presented. In contrast, Oostwoud Wijdenes et al. (2015) observed a larger SD of approximately 26° in the context of a 20° color change. It is possible that merging two color representations introduces substantial variability; it is also possible that the SD was overestimated in this study because the aggregate distribution reflected a mixture of two response distributions centered at the two original values.

A second candidate difference is that, in the present study, there was a strong demand to maintain separate pre- and post-saccadic representations, because the participants did not know until the post-cue which value to report. This method allowed us to draw strong conclusions about automatic overwriting, but it may have decreased the probability of integration. In contrast, Oostwoud Wijdenes et al. (2015) and Schut et al. (2018) instructed participants to report *the* color of the target despite the presentation of two different colors, which may have encouraged integration. (Demeyer et al., 2010b instructed participants to report the post-saccadic color on all trials, but there was no explicit demand to maintain two separate representations). If this were the critical methodological difference, then it would suggest that trans-saccadic color integration is under strategic control and can be avoided when dictated by task demands. In contrast, integration masking within a fixation, like all forms of masking, is automatic by definition: masking is a phenomenon observed in the context of attempting to maintain one representation and avoid interference from another. Thus, if trans-saccadic integration to form a merged representation is a robust phenomenon, it must operate in a functionally different manner than sensory integration within a fixation. Moreover, if this type of trans-saccadic integration were under strategic control, it would no longer provide a compelling explanation of insensitivity to trans-saccadic change. Change detection paradigms also introduce a strong demand to maintain separate representations for comparison, and if integration could be strategically avoided, participants could presumably avoid merging the two representations in change detection tasks as well. Thus, we think it is unlikely that the different results were driven by differences in task demands.

As we have been suggesting, a clear alternative is that, instead of reflecting a merged representation, the response distributions in Oostwoud Wijdenes et al. (2015), Schut et al. (2018), and Demeyer et al. (2010b) derived from a bivariate mixture of responses. Resolving this issue will likely require additional experimental work that bridges the methods and analytical techniques used here and in these three studies.

## Conclusion

The present results provide strong positive evidence in support of a trans-saccadic overwriting mechanism that is mediated by object continuity (Tas et al., 2012). First, when pre- and post-saccadic colors were likely to have been mapped to the same object representation, we observed substantial overwriting of the pre-saccadic color by the post-saccadic color. Second, the introduction of a post-saccadic blank period substantially improved pre-saccadic color report, consistent with protection from overwriting based on object discontinuity. In addition, the present results point to the need for further research to resolve the relative contributions of overwriting and integration in trans-saccadic perception.

## Supplementary Material

Supplement 1

## Footnotes

1 When the two patterns are incompatible, this process is referred to as *integration masking* (e.g., Enns & Di Lollo, 2000).

2 We use the terms *univariate* and *bivariate* to refer to the structure of responses (i.e., drawn from either one or two underlying distributions) and the terms *unimodal* and *bimodal* to refer to the resulting pattern in the aggregate distribution (one or two modes). The point here is that an underlying bivariate structure can often produce a unimodal distribution.

3 The two participants eliminated from the 15° sub-experiment had only 45% and 43% of trials retained after latency trimming (described below). The two participants eliminated from the 30° sub-experiment had only 50% and 38% of trials retained. Many of the individual conditions for these participants included fewer than 30 retained observations and thus fell below typical recommendations for mixture model analysis.

4 We conducted the same two analyses for the 15° and 30° sub-experiments. Although we cannot draw strong conclusions about overwriting from these data, it is important to ensure that they did not produce evidence that directly contradicted our predictions. These analyses were consistent with the analysis at 45°, both in terms of the direction of the effects and their statistical significance.

5 As discussed in the Introduction, Oostwoud Wijdenes et al. (2015) also found evidence for a reliability benefit when the pre- and post-saccadic stimuli were the same, indicating some form of transsaccadic integration. The question here is whether, in the experiments in which they presented different color values, there was sensory integration to form an intermediate value.
