# Supplementary material for: Object-mediated overwriting across saccades
Source: J Vis. 2021 Feb 4;21(2):3. doi: 10.1167/jov.21.2.3 (PMC7862732; doi:10.1167/jov.21.2.3)
Supplement: Supplement 1 [file jovi-21-2-3_s001.pdf]

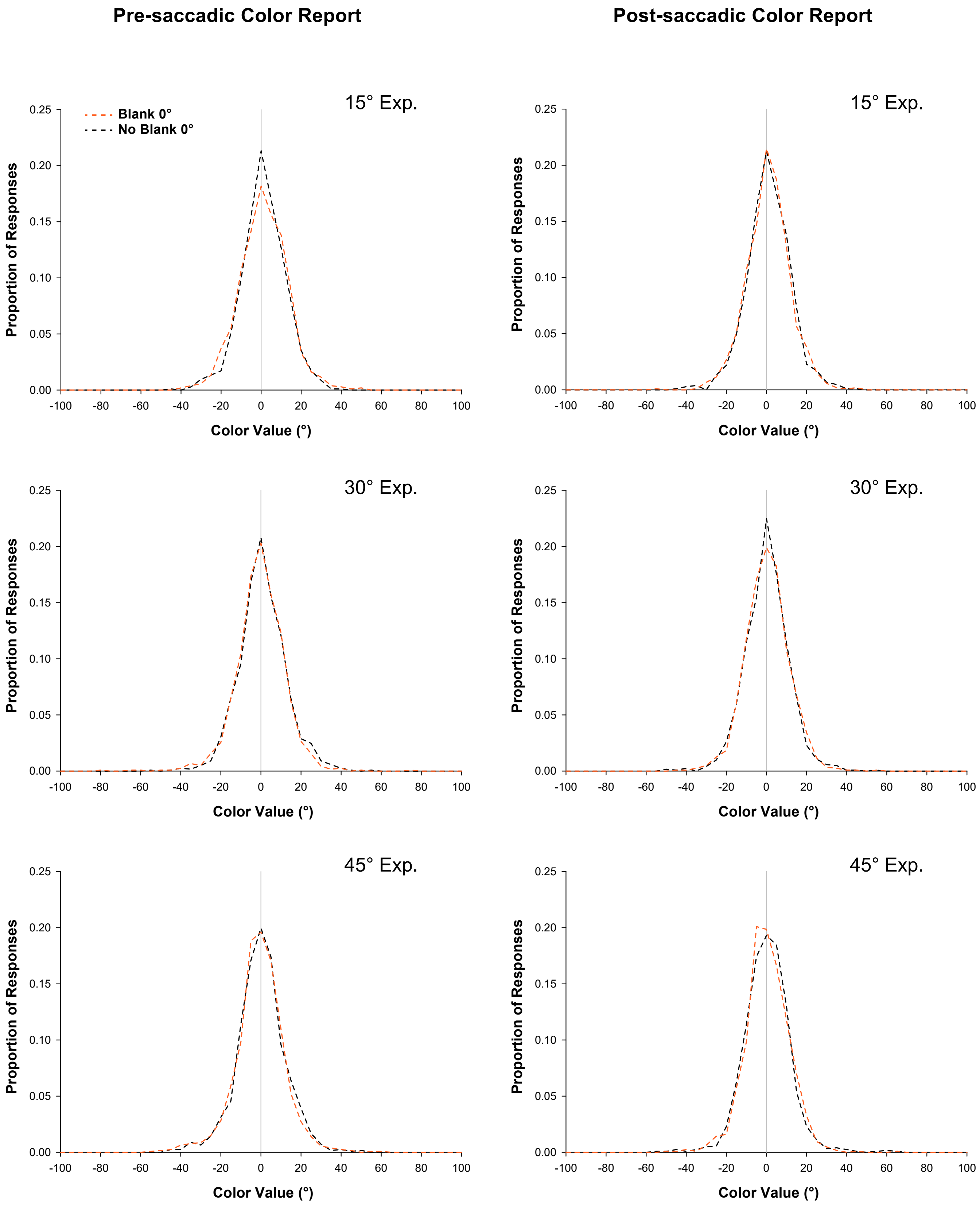

Supplementary Figure 1. No-change color response distributions for the Pre-report (left column) and Post-report (right column) trials as a function of color change magnitude and Blanking condition.
